# Discriminative possibilistic clustering promoting cross-domain emotion recognition

**DOI:** 10.3389/fnins.2024.1458815

**Published:** 2024-11-01

**Authors:** Yufang Dan, Di Zhou, Zhongheng Wang

**Affiliations:** ^1^Ningbo Polytechnic, Institute of Artificial Intelligence Application, Zhejiang, China; ^2^Industrial Technological Institute of Intelligent Manufacturing, Sichuan University of Arts and Science, Sichuang, China; ^3^Ningbo Vichnet Technology Co., Ltd., Zhejiang, China

**Keywords:** domain adaptation, probabilistic clustering, maximum mean discrepancy, fuzzy entropy, Electroencephalogram

## Abstract

The affective Brain-Computer Interface (aBCI) systems strive to enhance prediction accuracy for individual subjects by leveraging data from multiple subjects. However, significant differences in EEG (Electroencephalogram) feature patterns among subjects often hinder these systems from achieving the desired outcomes. Although studies have attempted to address this challenge using subject-specific classifier strategies, the scarcity of labeled data remains a major hurdle. In light of this, Domain Adaptation (DA) technology has gradually emerged as a prominent approach in the field of EEG-based emotion recognition, attracting widespread research interest. The crux of DA learning lies in resolving the issue of distribution mismatch between training and testing datasets, which has become a focal point of academic attention. Currently, mainstream DA methods primarily focus on mitigating domain distribution discrepancies by minimizing the Maximum Mean Discrepancy (MMD) or its variants. Nevertheless, the presence of noisy samples in datasets can lead to pronounced shifts in domain means, thereby impairing the adaptive performance of DA methods based on MMD and its variants in practical applications to some extent. Research has revealed that the traditional MMD metric can be transformed into a 1-center clustering problem, and the possibility clustering model is adept at mitigating noise interference during the data clustering process. Consequently, the conventional MMD metric can be further relaxed into a possibilistic clustering model. Therefore, we construct a distributed distance measure with Discriminative Possibilistic Clustering criterion (DPC), which aims to achieve two objectives: (1) ensuring the discriminative effectiveness of domain distribution alignment by finding a shared subspace that minimizes the overall distribution distance between domains while maximizing the semantic distribution distance according to the principle of “sames attract and opposites repel”; and (2) enhancing the robustness of distribution distance measure by introducing a fuzzy entropy regularization term. Theoretical analysis confirms that the proposed DPC is an upper bound of the existing MMD metric under certain conditions. Therefore, the MMD objective can be effectively optimized by minimizing the DPC. Finally, we propose a domain adaptation in Emotion recognition based on DPC (EDPC) that introduces a graph Laplacian matrix to preserve the geometric structural consistency between data within the source and target domains, thereby enhancing label propagation performance. Simultaneously, by maximizing the use of source domain discriminative information to minimize domain discrimination errors, the generalization performance of the DA model is further improved. Comparative experiments on several representative domain adaptation learning methods using multiple EEG datasets (i.e., SEED and SEED-IV) show that, in most cases, the proposed method exhibits better or comparable consistent generalization performance.

## 1 Introduction

Within the research domain of affective computing (Mühl et al., 2014), Automatic Emotion Recognition (AER; Dolan, 2002) has garnered extensive interest and attention from researchers in the field of computer vision (Kim et al., 2013; Zhang et al., 2017). To date, numerous emotion recognition methods based on Electroencephalogram (EEG) have been successively proposed (Zheng, 2017; Li X. et al., 2018; Pandey and Seeja, 2019; Jenke et al., 2014; Musha et al., 1997). From the perspective of machine learning, EEG-based AER tasks can be formulated as classification or regression problems for processing (Kim et al., 2013; Zhang et al., 2017). In such tasks, state-of-the-art AER techniques often involve training classifiers on data from multiple subjects to achieve precise emotion recognition. However, classifiers that rely on specific subjects typically exhibit limited generalization capabilities due to significant variations in emotional expression patterns across different subjects (Pandey and Seeja, 2019). By optimizing feature representations and learning models (Li et al., 2018a, 2018b, 2019; Du et al., 2020; Song et al., 2018; Zhong et al., 2020; Zheng and Lu, 2015a, Zheng et al., 2015b), the accuracy of emotion recognition has been significantly improved. Given the inherent individual differences in EEG-based AER, applying the learned classifiers to unseen subjects may yield unsatisfactory results based on qualitative and empirical observations (Ghifary et al., 2017; Lan et al., 2018; Jayaram et al., 2016; Zheng and Lu, 2016; Wang et al., 2022). To address this issue, one potential solution is to adopt subject-specific classifiers, but this approach is often impractical due to the scarcity of training data. Furthermore, even if this approach is feasible in certain scenarios, fine-tuning the classifier to maintain its good recognition performance is indispensable, partly because EEG signals from the same subject can change over time (Zhou et al., 2022). To tackle these challenges, the Domain Adaptation (DA) learning paradigm has emerged and has been widely and effectively applied (Patel et al., 2015; Dan et al., 2022; Tao et al., 2017, 2021, 2022; Zhang Y. et al., 2019). This paradigm aims to enhance the learning performance of the target domain (where labeled samples are scarce or absent) by transferring and leveraging prior knowledge from other related but differently distributed domains (i.e., source or auxiliary domains).

To achieve effective knowledge transfer across different professional domains, the crux lies in ensuring similarity or consistency in data distributions between the source domain and the target domain. Since the discrepancies in data distributions are particularly pronounced and more complex in sentiment analysis and other emotionally related fields, a significant challenge is posed for Domain Adaptation (DA) learning at present. Currently, a commonly adopted approach in the field of DA learning is to address distribution differences by identifying features (or samples) that remain constant across different domains (Pan and Yang, 2010; Patel et al., 2015). In order to more effectively leverage these domain-invariant features, traditional shallow DA models have gradually evolved into deep DA models. These deep DA models (Long et al., 2015, 2016; Chen et al., 2019; Lee et al., 2019; Ding et al., 2018; Tang and Jia, 2019) with their profound feature transformation capabilities have made notable advancements in adaptive learning. Although deep DA models can mitigate the impact of distribution differences between domains when dealing with large datasets, they have not yet fully resolved the issue of domain shift. Deep DA methods exhibit robust performance. The specific mechanisms underlying these effects remain unclear, since these advantages may stem from various factors such as deep feature representations, model fine-tuning, or adaptive regularization. More importantly, the learning outcomes of these methods still lack adequate explanation and validation at both theoretical and practical levels.

To better characterize the generalization capability of shallow DA algorithms, existing theoretical research on DA has proposed [i.e., the generalization error bound (Ben-David et al., 2010)] for DA by the following inequality:


(1) $$\begin{array}{l} e_{T}\left(h\right)\leq e_{S}\left(h\right)+d_{H}\left(D_{S},D_{T}\right)+\\ \;\min\left\{\begin{array}{l} \varepsilon_{D_{S}}\left[\left|f_{S}\left(x\right)-f_{T}\left(x\right)\right|\right],\\ \varepsilon_{D_{T}}\left[\left|f_{S}\left(x\right)-f_{T}\left(x\right)\right|\right] \end{array}\right\} \end{array}$$

Based on Equation 1, it can be seen that the upper bound of the expected error $e_{T}\left(h\right)$ of the target hypothesis h is mainly determined by three aspects: the expected error $e_{S}\left(h\right)$ of the source domain hypothesis, the distribution discrepancy $d_{H}\left(D_{S},D_{T}\right)$ between the source and target domains, and the discrepancy in the labeling functions between the two domains. Therefore, to reduce the DA generalization error, in addition to retaining the discriminative information of the source domain (the first aspect), it is also necessary to consider reducing the distribution discrepancy between domains (the second aspect) and the discrepancy in the labeling functions between domains (the third aspect). Accordingly, current mainstream DA methods can be divided into those based on distribution alignment (including instance weighting and feature transformation) and those based on classifier model alignment (Gretton et al., 2007; Chu et al., 2013; Pan et al., 2011; Long et al., 2013; Luo et al., 2020; Baktashmotlagh et al., 2013; Ganin et al., 2016; Kang et al., 2022; Liang et al., 2019; Tao et al., 2012; Tao et al., 2015; Tao et al., 2016; Tao et al., 2019).

To address the challenges posed by domain distribution shift, early research endeavors adopted an instance weighting strategy. This strategy involves calculating the probability of each sample belonging to either the source or target domain, referred to as the instance’s membership weight, and subsequently mitigating the domain shift issue by re-weighting the samples. Among these techniques, the Maximum Mean Discrepancy (MMD; Gretton et al., 2007) has been widely applied due to its simplicity and effectiveness. However, its optimization process is often isolated from the training of the classifier, making it difficult to achieve simultaneous optimization of both. In response to this limitation, Chu et al. (2013) proposed a domain-adaptive classifier that integrates instance weighting. In order to further transcend the constraints imposed by the assumption of conditional distribution consistency in instance weighting methods, feature transformation methods have become a focal point of research in recent years (Pan et al., 2011; Long et al., 2013; Luo et al., 2020; Baktashmotlagh et al., 2013; Kang et al., 2022; Liang et al., 2019). For instance, Pan et al. (2011) introduced the Transfer Component Analysis (TCA) method. This approach aims to minimize the MMD distance between the source and target domain distributions by learning a transformation matrix while preserving the original variance of the data. Unfortunately, it does not take into account the semantic consistency alignment across different domains. To address this, Long et al. (2013) proposed the Joint Domain Adaptation (Joint DA, JDA) method, which not only considers feature distribution alignment but also accounts for conditional distribution alignment, and initializes the category labels of the target domain using pseudo-labels. More recently, Luo et al. (2020) presented a unified domain adaptation framework, DGA-DA, that synthesizes the ideas of TCA and JDA. This framework introduces a strategy of inter-domain dissimilar exclusion and retains the geometric structure information of domain data, thereby effectively facilitating the propagation of target labels. Most existing affective models are based on deep transfer learning methods utilizing domain-adversarial neural networks (DANN; Ganin et al., 2016), as seen in studies (Li et al., 2018c; Li et al., 2018d; Du et al., 2020; Luo et al., 2018; Sun et al., 2022). DANN aims to find a shared feature representation for both source and target domains with indistinguishable distribution differences while maintaining the predictive capacity of the features on source samples for specific classification tasks. Additionally, the framework preserves the geometric structure information of domain data to ensure effective target label propagation. Baktashmotlagh et al. (2013) introduced the Domain Invariant Projection (DIP) algorithm. This algorithm applies a polynomial kernel to the MMD metric, aiming to construct a compact shared feature space and minimize the intra-class scatter through a clustering-based method.

A comprehensive review of the current state of Domain Adaptation (DA) research reveals that Maximum Mean Discrepancy (MMD) is a widely employed measure of distribution distance in the field of feature transformation. Traditional MMD-based DA methods primarily focus on reducing the distribution discrepancies between different domains. However, these methods often overlook the statistical (clustering) structure of the target domain data, which can adversely affect the inference of target domain labels. To address this issue, Kang et al. (2022) proposed an unsupervised DA method known as the Contrastive Adaptation Network. This approach hypothesizes the labels of the target domain through clustering and utilizes contrastive difference metrics in multiple fully connected layers to adjust the feature representations, aiming to minimize intra-class domain differences and maximize inter-class domain differences. During the training process, the hypotheses of target labels and feature representations are iteratively optimized in a cross-manner to enhance the model’s generalization capability. Concurrently, inspired by clustering methods, Liang et al. (2019) developed an effective Domain Invariant Projection Ensemble method. This method leverages clustering principles to seek the optimal projections among various categories within the domain, thereby narrowing the semantic gap between domains and enhancing the cohesion of intra-domain categories. Nevertheless, these methods essentially remain within the scope of MMD-based feature transformation DA methods.

It is worth noting that existing MMD-based methods did not fully consider the impact of intra-domain noise when measuring domain distribution distance. In real scenarios, noise inherently exists in domains. The intra-domain noise can lead to mean-shift problems on distance measure by traditional MMD methods and their variants. This phenomenon to some extent is affecting the generalization performance of MMD-based DA methods (Tao et al., 2023).

Fortunately, the proposed possibilistic clustering models (Krishnapuram and Keller, 1993; Dan et al., 2021) offer a comprehensive solution to the aforementioned issues. Unlike hard clustering models, possibilistic clustering effectively suppresses noise interference during the data clustering process (Dan et al., 2021). Inspired by this, the traditional MMD metric is relaxed into one-center center objective to tackle the mean shift problem of domain distributions in noisy environments. The possibilistic one-center center model with a fuzzy entropy regularization term is reconstructed. Then, we propose a distributed distance measure with Discriminative Possibilistic Clustering (DPC) criterion, and further develop a domain adaptation in Emotion recognition based on DPC model (EDPC). EDPC mainly comprises two jointly optimized components: DPC and classifier. First, DPC seeks a shared latent space to achieve discriminative alignment of overall domain distribution and inter-domain semantic distribution, the samples adhering to the principle of attract each other with similar class and repulse each other with different class. Clustering membership is adopted to indicate the likelihood of each sample being consistent with the overall domain distribution. It aims to suppress the impact of noisy data during domain matching and enhance the robustness and effectiveness of domain distribution clustering metrics. Second, in the classifier model learning phase, a graph Laplacian regularization term is introduced to preserve the local geometric structure of sample in the latent domain space to improve the performance of domain label propagation. Finally, we gain superior knowledge transfer performance by maximizing the utilization of source domain discriminative information minimizing the discriminative error in the target domain. The EDPC method will be evaluated in emotion recognition. Compared to other state-of-the-art methods, this approach demonstrates significant performance improvements in most cases. The major novelties of the proposed EDPC are summarized as follows:

1. To address the noisy problem in existed methods, we establish a robust distributed distance measure with Possibilistic Clustering by relaxing the traditional MMD metric criterion into a possibilistic one-center center model. From a theoretical point of view, we proposed measure method serves as an upper bound to the traditional MMD metric under certain conditions.
2. The samples follow the principle of “attract each other with similar class and repulse each other with different class,” we extend and establish a robust distributed distance measure with Discriminative Possibilistic Clustering (i.e., DPC) metric criterion. Based on this, we seek to optimize a domain-invariant subspace that achieves joint alignment of overall domain distribution and inter-domain semantic distribution.
3. We propose a unified domain adaptation Emotion recognition model based on DPC (i.e., EDPC). An iterative optimization algorithm is provided. We finally prove its consistent convergence and propose a generalization error bound for the model based on Rademacher complexity theory.
4. Extensive experiments on six real-world datasets validate the robust effectiveness of the proposed method.

## 2 Proposed method

In DA learning, the source domain is defined as $D_{S}={\left\{x_{i}^{s},y_{i}^{s}\right\}}_{i=1}^{n}$ , where the sample set is defined as $X^{s}=\left[x_{1}^{s},\ldots ,x_{n}^{s}\right]\in {\mathbb{R}}^{d\times n}$ , and the corresponding class labels are defined as $Y^{s}={\left\{y_{1},\ldots ,y_{n}\right\}}^{T}\in {\left\{0,1\right\}}^{n\times C}$ . Here, $Y^{s}={\left\{y_{1},\ldots ,y_{n}\right\}}^{T}\in {\left\{0,1\right\}}^{n\times C}$ is a one-hot encoded vector; if ${\hat{x}}_{i}$ belongs to the j -th class, then $y_{i}^{j}=1$ . The unlabeled target domain is defined as $D_{T}={\left\{{\hat{x}}_{j}^{t}\right\}}_{j=1}^{m}$ , where the sample set and unknown sample labels during training are ${\hat{X}}^{t}=\left[{\hat{x}}_{1}^{t},\ldots ,{\hat{x}}_{m}^{t}\right]\in {\mathbb{R}}^{d\times m}$ , $Y^{t}={\left\{y_{1},\ldots ,y_{m}\right\}}^{T}\in {\mathbb{R}}^{m\times C}$ , respectively. We further define $\hat{X}=\left[{\hat{X}}^{s},{\hat{X}}^{t}\right]\in {\mathbb{R}}^{d\times N}$ and $Y=\left[Y^{s},Y^{t}\right]\in {\mathbb{R}}^{N\times C}$ , where N=n+m . Let $\mu_{s}$ and $\mu_{t}$ be the mean values of the samples in the source and target domains, respectively. Our work has the following assumptions:

1. The source domain distribution ℙ and the target domain distribution ℚ are different, but they share the same feature space X , i.e., $\mathbb{P}\left(X_{S}\right)\neq \mathbb{Q}\left(X_{T}\right)$ and $X_{S}=X_{T}$ , where $X_{S},X_{T}\in X$ are the feature spaces of the source domain and the target domain, respectively.
2. The class-conditional probability distributions between domains are different but they share the same label space Y , i.e., $\mathbb{P}\left(Y_{S}|X_{S}\right)\neq \mathbb{Q}\left(Y_{T}|X_{T}\right)$ , where $Y_{S},Y_{T}\in Y$ are the label spaces of the source domain and the target domain, respectively.

The rest of this section is organized as follows. In Subsection 2.1, we give the general formulation of the proposed method. We describe the details of the general formulation in Subsection 2.2 about discriminative possibilistic clustering (i.e., DPC) formulation and Subsection 2.3 about the classifier in emotion recognition with DPC. The final detail formulation is showed in Subsection 2.4.

### 2.1 General formulation

To effectively align domains’ distribution and achieve maximum knowledge transferring, this paper seeks to optimize a domain-invariant subspace $P\in {\mathbb{R}}^{d\times r}$ , where r is the subspace dimension and making P be an orthogonal subspace (i.e., $P^{T}P=I$ , $I\in {\mathbb{R}}^{r\times r}$ is the identity matrix). In other words, Our work is to achieve $\mathbb{P}\left(P\left(X_{S}\right)\right)\approx \mathbb{Q}\left(P\left(X_{T}\right)\right)$ and $\mathbb{P}\left(Y_{S}\left|P\left(X_{S}\right)\right.\right)\approx \mathbb{Q}\left(Y_{T}\left|P\left(X_{T}\right)\right.\right)$ in the optimized domain-invariant subspace P .

For the problem of DA in complex structures and noisy environments, we aim to improve the robustness of distribution distance metrics for DA and enhance generalization in the target domain. Based on the DA generalization error theory (Ben-David et al., 2010), we explore to achieve the following two core objectives: First, we construct a robust distribution distance metric that can resist the impact of noise for addressing the issue of domain mean-shift. The differences of domains distribution can be selectively corrected. Second, we effectively perform semantic reasoning in the target domain by maintaining the geometric structure consistency of samples in domain and connecting the discriminative information of the source domain and minimizing the discriminative error in the target domain. The a highly generalizable target domain classifier be constructed. Therefore, our general framework can be described as following:


(2) $$\begin{array}{l} \Theta \left(\lambda ,P,Y^{t},W\right)=\min R\left(f_{W}\left(P\left(\hat{X}\right)\right),Y\right)\\ +\alpha \Omega_{\lambda }\left(P\left({\hat{X}}^{s}\right),\;P\left({\hat{X}}^{t}\right)\right)-\beta \Omega_{\mathrm{rep}}\left(P\left({\hat{X}}^{s}\right),\;P\left({\hat{X}}^{t}\right)\right)\\ s.t.P^{T}P=I \end{array}$$

where the first term is the empirical risk function of the decision function $f_{W}\left(P\left(\hat{X}\right)\right)$ on the domain sample, W represents the model parameters, and $R\left(\text{.}\right)$ is the empirical loss function. It can be chosen according to different application needs. The second term is a regularization term that measures the semantic distribution discrepancy between $D_{S}$ and $D_{T}$ in the domain-invariant subspace P . λ denotes the clustering membership degree. The last term aims to enhance the discriminative ability for the second term. The orthogonal constraint $P^{T}P=I$ serves as a regularization constraint on P . It does not need to be described in the objective function. Two hyper-parameters (i.e., α≥0 and β≥0 ) are used to measure the semantic distribution discrepancy and the inter-classes distribution discrepancy of the two domains, respectively. Therefore, the subspace P , the membership degree λ , and the decision function $f_{W}\left(\cdot \right)$ can be learned simultaneously during optimizing the Problem 2.

### 2.2 Discriminative possibilistic clustering formulation

The rest of this subsection is organized as follows. In 2.2.1, we give the motivation of the proposed method DPC and proof the MMD metric can be relaxed and modeled as a special one-center clustering problem. We then explain all details of DPC metric in 2.2.2.

#### 2.2.1 Motivation

In a certain Reproducing Kernel Hilbert Space (RKHS) H , the original space data is transformed into feature representations in the RKHS through a nonlinear mapping $\phi :{\mathbb{R}}^{d}\to H$ . The corresponding kernel function is defined as $K\left(.,.\right):\hat{X}\times \hat{X}\to \mathbb{R}$ , where $K\left({\hat{x}}_{1},{\hat{x}}_{2}\right)=\phi \left({\hat{x}}_{1}\right),\phi {\left({\hat{x}}_{2}\right)}_{H}$ , ${\hat{x}}_{1},{\hat{x}}_{2}\in \hat{X}$ . Here, $\phi \left(\hat{x}\right)$ is a feature mapping function that maps samples from the original space to a high-dimensional or even infinite-dimensional space (i.e., the RKHS H ). For uniform representation, the mapping result is defined as following:


$$x=\left\{\begin{array}{c} \hat{x},\;\mathrm{linear kernel mapping}\\ \begin{array}{cc} k\left(\hat{x},.\right), & non-\mathrm{linear kernel mapping} \end{array} \end{array}\right.$$

where $k\left(\hat{x},.\right)={\left[k\left(\hat{x},{\hat{x}}_{1}\right),k\left(\hat{x},{\hat{x}}_{2}\right),\ldots ,k\left(\hat{x},{\hat{x}}_{n}\right)\right]}^{T}$ is called the empirical kernel mapping [32]. Then $X^{s}$ and $X^{t}$ respectively represent the unified representation of linear kernel mapping and nonlinear kernel mapping of ${\hat{X}}^{s}$ and ${\hat{X}}^{t}$ . Accordingly, $X=\left[X^{s},X^{t}\right]$ .

Therefore, we try to learn a DA learning machine $f\left(\phi \left(\hat{x}\right)\right)=W^{'T}\phi \left(\hat{x}\right)$ . Thus, the formal description of Problem 2 can be rewritten in Equation (3):


(3) $$\begin{array}{l} \Theta \left(\lambda ,P,Y^{t},W\right)=\min R\left(f_{W}\left(P\left(X\right)\right),Y\right)+\alpha \Omega_{\lambda }\left(P\left(X^{s}\right),\;P\left(X^{t}\right)\right)\\ \;\;-\beta \Omega_{\mathrm{rep}}\left(P\left(X^{s}\right),\;P\left(X^{t}\right)\right)\\ s.t.P^{T}P=I \end{array}$$

In classical DA research, a commonly adopted strategy to address the domain mean-shift problem is to reduce the distribution distance between the source domain and the target domain in a certain latent feature space. This involves identifying a feature descriptor space, where MMD is a frequently used method to measure the distribution difference between two domains. MMD utilizes the framework of RKHS to effectively quantify the gap between two distributions [as described in references (Bruzzone and Marconcini, 2010; Gretton et al., 2010)]. It is assumed that there exists a set G containing all domain-invariant transformation matrices P (i.e., $G==\left\{P\in {\mathbb{R}}^{r}|P^{T}P=I\right\}$ ) in this framework. The maximum empirical mean discrepancy between the source domain distribution ℙ and the target domain distribution ℚ can be defined in Equation (4):


(4) $$MMD_{G}\left(X^{s},X^{t}\right)={\left\|\frac{1}{n}\overset{n}{\underset{i=1}{\sum }}P\left(x_{i}^{s}\right)-\frac{1}{m}\overset{m}{\underset{j=1}{\sum }}P\left(x_{j}^{t}\right)\right\|}_{H}^{2}$$

According to Hoeffding’s inequality theorem, when the domain sample size is sufficiently large (or approaches infinity), the expected difference and the empirical mean difference are approximately (or equal). To illustrate the generalized connection between the traditional MMD criterion and the mean clustering model, the following theorem is presented:

**Theorem 1.** The MMD metric can be relaxed and modeled as a special one-center clustering problem, where the clustering center of the one-center is μ and the sample clustering membership is the vector ς .

**Proof:** From the definition of empirical MMD, we have (let $X\leftarrow P\left(X\right)$ for simplify):


(5) $$\begin{array}{l} MMD\left(X^{s},X^{t}\right)\\ ={\left\|\frac{1}{n}\sum_{i=1}^{n}x_{i}^{s}-\frac{1}{m}\sum_{j=1}^{m}x_{j}^{t}\right\|}_{H}^{2}\\ ={\left\|\frac{1}{n}\sum_{i=1}^{n}x_{i}^{s}-\mu +\mu -\frac{1}{m}\sum_{j=1}^{m}x_{j}^{t}\right\|}_{H}^{2}\\ \leq {\left\|\frac{1}{n}\sum_{i=1}^{n}x_{i}^{s}-\mu \right\|}_{H}^{2}+{\left\|\frac{1}{m}\sum_{j=1}^{m}x_{j}^{t}-\mu \right\|}_{H}^{2}\\ =\frac{1}{n^{2}}{\left\|\sum_{i=1}^{n}x_{i}^{s}-n\mu \right\|}_{H}^{2}+\frac{1}{m^{2}}{\left\|\sum_{j=1}^{m}x_{j}^{t}-m\mu \right\|}_{H}^{2}\\ =\frac{1}{n^{2}}{\left\|\sum_{i=1}^{n}\left(x_{i}^{s}-\mu \right)\right\|}_{H}^{2}+\frac{1}{m^{2}}{\left\|\sum_{j=1}^{m}\left(x_{j}^{t}-\mu \right)\right\|}_{H}^{2}\\ \leq \frac{1}{n^{2}}{\sum_{i=1}^{n}\left\|x_{i}^{s}-\mu \right\|}_{H}^{2}+\frac{1}{m^{2}}\sum_{j=1}^{m}{\left\|x_{j}^{t}-\mu \right\|}_{H}^{2}\\ =\sum_{k=1}^{N}\varsigma_{k}{\left\|x_{k}-\mu \right\|}_{H}^{2} \end{array}$$

where the cluster center is defined as $\mu =\delta \mu_{s}+\left(1-\delta \right)\mu_{t}$ , 0≤δ≤1 ; when *n* = *m*,^1^ let δ=0.5 ; the sample membership of the one-center center $\varsigma ={\left\{\varsigma_{k}\right\}}_{k=1}^{N}$ is defined in Equation (6):


(6) $$\varsigma_{k}=\{\begin{array}{l} \frac{1}{n^{2}},\;x_{k}\in X^{s}\\ \frac{1}{m^{2}},\;x_{k}\in X^{t} \end{array}$$

By analyzing Equation 5, it can be inferred that the one-center is μ actually constitutes an upper bound of the traditional MMD metric. It means that the MMD metric can be simplified to a special form of the one-center objective function. In this case, it is possible to minimize the MMD between different domains by optimizing this special clustering objective.

According to Theorem 1 and the explanation in Baktashmotlagh et al. (2013), it can be recognized that the MMD metric standard for domain distribution is essentially related to the clustering model. It is possible to more effectively achieve distribution alignment between different domains promote domain adaptive learning by clustering domain data. It should be noted that traditional clustering models are usually sensitive to noise (Krishnapuram and Keller, 1993), which limits MMD-based DA methods and make them prone to domain mean-shift issues in noisy environment. To address this challenge, we further explore more robust clustering methods and propose a new discriminative domain distribution distance metric criterion in following subsection.

#### 2.2.2 Discriminative possibilistic clustering

##### 2.2.2.1 Possibilistic clustering

Recently proposed probabilistic clustering methods have been proven to effectively mitigate the negative impact of noise on clustering results (Dan et al., 2021). In light of this, this section extends the original one-center method to the realm of probabilistic one-center. Then we propose a Possibilistic Clustering distribution distance metric in domain-invariant subspace P , namely PC. We extends the hard clustering approach of MMD to a soft clustering form by incorporating the concept of possibilistic clustering entropy. In this framework, each sample determines its contribution based on its distance from the overall domain mean (that is, the greater the distance of the data sample, the lower its contribution, and conversely, the more likely it is considered as noise). In this way, PC allows for the attenuation of the impact of mean-shift caused by noise during domain alignment through adjustment. Therefore, the formula for the possibilistic clustering distribution distance metric can be defined as follows:


(7) $$\begin{array}{l} PC\left(X^{s},X^{t},\lambda \right)=\overset{N}{\underset{k=1}{\sum }}\lambda_{k}^{b}{\left\|P\left(x_{k}\right)-P\left(\mu \right)\right\|}_{H}^{2}\\ s.t.,0\leq \lambda_{k}\leq 1,k=1,\ldots ,N \end{array}$$

where $\lambda =\left[\lambda_{1},\ldots ,\lambda_{N}\right]$ is the possibilistic clustering membership vector, the parameter b is the weight exponent of $\lambda_{k}$ is used to adjust the uncertainty or degree of samples belong to multiple categories. To avoid trivial solutions, we set b=2 in the subsequent formulas. The detailed introduction to different values of b is referred to (Krishnapuram and Keller, 1993). According to Equation 7, $PC\left(X^{s},X^{t},\lambda \right)$ is a possibilistic one-center objective function (with the cluster center being μ ). When $\lambda_{k}^{2}=\varsigma_{k}$ , $PC\left(X^{s},X^{t},\lambda \right)$ represents the aforementioned special form of one-center. Next, we verify that the proposed $PC\left(X^{s},X^{t},\lambda \right)$ is an upper bound of the traditional MMD metric at a certain condition by the following theorem:

**Theorem 2.** When the possibilistic clustering membership satisfies $\lambda_{k}\in \left[\frac{1}{r},1\right]$ ( k=1,…,N ), the possibilistic distribution distance metric $PC\left(X^{s},X^{t},\lambda \right)$ is an upper bound of the traditional MMD metric.

**Proof:** Combining Equations 5 and 7, we can obtain:


(8) $$\begin{array}{l} MMD\left(X^{s},X^{t}\right)\\ \leq \overset{N}{\underset{k=1}{\sum }}\varsigma_{k}{\left\|P\left(x_{k}\right)-P\left(\mu \right)\right\|}_{H}^{2}\\ \leq \overset{N}{\underset{k=1}{\sum }}\lambda_{k}^{2}{\left\|P\left(x_{k}\right)-P\left(\mu \right)\right\|}_{H}^{2}\\ =PC\left(X^{s},X^{t},\lambda \right) \end{array}$$

According to the value range of $\varsigma_{k}$ , when λk∈1r1 and $r=\min\left(n,m\right)$ , the second inequality in Equation 8 holds true and the conclusion is proved.

Guided by Theorem 1 and Theorem 2, we are able to reconstruct the traditional MMD metric into a function that targets 1-center probabilistic clustering. From this new perspective, we can gain a profound understanding that the objective of probability distribution distance measure is not only to facilitate effective adjustment of feature distributions across different domains but also to mitigate the negative transfer effects induced by intra-domain noisy data during the training process.

In the PC described in Equation 7, its primary objective is centered on reducing the statistical distribution discrepancy between the source domain and the target domain. However, this method fails to adequately emphasize the importance of preserving the semantic structural information of instances during the process of domain distribution matching. This approach may compromise the ability to distinguish between categories within the domains. To preserve the distinctiveness of the statistical distribution structures between domains, we further propose a Discriminative PC model (i.e., DPC). This model aims to implement discriminative statistical distribution alignment between the source and target domains while adjusting the semantic distribution through a clustering hypothesis based on likelihood.

The samples adhere to the principle of “attract each other with similar class and repulse each other with different class.” DPC criterion aims to achieve dual objectives: first, it reduces the distribution bias of similar samples within different domains, thereby minimizing semantic differences between domains; second, it increases the distribution gap between different classes of samples within different domains by enhancing the differentiability of domain samples.

##### 2.2.2.2 DPC with Intra-class alignment

In the PC model shown in Equation 7, the process of domain distribution alignment does not consider the semantic structure information of samples. This oversight may compromise the local discriminative structure between different categories within the domain. To address this issue, Tao et al. (2016) proposes further considering the semantic distribution structure between domains during the alignment process and evaluating the contribution of each sample to semantic matching. Hence, we have the following DPC framework with semantic registration functionality by extending the PC model:


(9) $$\begin{array}{l} \Omega_{\lambda }^{'}\left(P\left(X^{s}\right),\;P\left(X^{t}\right)\right)\\ =\min_{\lambda_{k,c}}\overset{C}{\underset{c=0}{\sum }}\overset{N}{\underset{k=1}{\sum }}\lambda_{k,c}^{2}{\left\|P^{T}x_{k,c}-P^{T}\mu_{c}\right\|}_{H}^{2}\\ s.t.,0\leq \lambda_{k,c}\leq 1 \end{array}$$

where $\mu_{c}=\delta \mu_{s,c}+\left(1-\delta \right)\mu_{t,c}$ , $\mu_{s,c}=\frac{1}{n}\overset{C}{\underset{c=0}{\sum }}\overset{n_{c}}{\underset{i=1}{\sum }}{\tilde{x}}_{i,c}^{s}$ , $\mu_{t,c}=\frac{1}{m}\overset{C}{\underset{c=0}{\sum }}\overset{m_{c}}{\underset{j=1}{\sum }}x_{j,c}^{t}$ , c=0,1,2,…,C , C is the number of classes in the target domain, $n_{c}$ is the number of samples in the c−th class of the source domain, $m_{c}$ is the number of samples in the c−th class of the target domain, $n=\overset{C}{\underset{c=0}{\sum }}n_{c}$ , and $m=\overset{C}{\underset{c=0}{\sum }}m_{c}$ . When c=0 , $\mu_{s,c}$ and $\mu_{t,c}$ represent the mean of the entire source domain and the entire target domain, respectively. Equation 9 is the form of feature distribution alignment. When $c\in \left[1,2,\ldots ,C\right]$ , $\mu_{s,c}$ and $\mu_{t,c}$ represent the mean of the corresponding classes in the source domain and target domain, respectively. $\lambda_{k,c}$ is the matching contribution value of $x_{k}$ belonging to the c−th category in the domain.

To enhance the robustness and effectiveness of the possibilistic clustering distribution distance measure method in handling noisy data, a fuzzy entropy regularization term, which is related to the parameter $\lambda_{k,c}$ , is introduced based on Equation 9. With this improvement, the DPC for semantic alignment can be redefined as following:


(10) $$\begin{array}{l} \Omega_{\lambda }\left(P\left(X^{s}\right),\;P\left(X^{t}\right)\right)\\ =\overset{C}{\underset{c=0}{\sum }}\overset{N}{\underset{k=1}{\sum }}\lambda_{k,c}^{2}{\left\|P^{T}x_{k,c}-P^{T}\mu_{c}\right\|}_{H}^{2}\\ +\beta \overset{C}{\underset{c=0}{\sum }}\overset{N}{\underset{k=1}{\sum }}\left(\lambda_{k,c}^{2}\ln\lambda_{k,c}^{2}-\lambda_{k,c}^{2}\right)\\ s.t.,0\leq \lambda_{k,c}\leq 1 \end{array}$$

where the parameter β serves as an adjustable balancing factor, aimed at ensuring that the value of the relevant data $\lambda_{k,c}$ remains at a high level to avoid obtaining trivial solutions that lack discriminative power. The improved DPC model now becomes a function that monotonically decreases as the value of $\lambda_{k,c}$ decreases. This model uses the second term in Equation 10, namely fuzzy entropy, to mitigate the adverse impact of noisy data on the model’s classification decisions. An increase in fuzzy entropy signifies an enhancement in the discriminatory information content of samples, which plays a positive role in strengthening the robust effectiveness of distribution distance measures. Furthermore, the introduction of a fuzzy entropy-regularized possibility distribution distance measure model can effectively limit the influence of noisy data in domain distribution alignment, reducing the interference of noise or outlier data in the domain adaptation learning process. For more details and empirical analysis on how fuzzy entropy improves robustness, refer to the discussion in reference (Gretton et al., 2010).

##### 2.2.2.3 DPC with Inter-class discrimination

The intra-class alignment neglects inter-class discrimination. We therefore add an additional inter-class repulsion term into the DPC model to increase the inter-class distance across domain. It enhances the semantic discriminatory of samples in the domain-invariant subspace and improves the robustness and effectiveness of domain adaptation learning. Specifically, let $\Omega_{\mathrm{rep}}\left(P\left(X^{s}\right),P\left(X^{t}\right)\right)$ be the inter-domain different classes repulsion term, which is defined as the total difference between the mean of each class $L^{c}$ and the mean of all other classes $L^{\left(\mathrm{cc}\right):\mathrm{cc}\in \left\{\left\{1,\ldots ,C\right\}-\left\{c\right\}\right\}}$ (excluding class *c*). That is:


(11) $$\begin{array}{l} \Omega_{\mathrm{rep}}\left(P\left(X^{s}\right),P\left(X^{t}\right)\right)\\ =\overset{C}{\underset{c=1}{\sum }}{\left\|\frac{1}{nc}\underset{x_{i}\in L^{\left(c\right)}}{\sum }P^{T}x_{i}-\frac{1}{\underset{\mathrm{cc}}{\sum }n_{\mathrm{cc}}}\underset{x_{j}\in L^{\left(\mathrm{cc}\right)}}{\sum }P^{T}x_{j}\right\|}^{2}\\ =\overset{C}{\underset{c=1}{\sum }}tr\left(P^{T}XM_{\mathrm{cc}}X^{T}P\right) \end{array}$$

where,


$$M_{\mathrm{cc}}=\{\begin{array}{l} \frac{1}{n_{c}n_{c}},\;\;x_{i},x_{j}\in L^{\left(c\right)}\\ \frac{1}{n_{\mathrm{cc}}n_{\mathrm{cc}}},\;\;x_{i},x_{j}\in L^{\left(\mathrm{cc}\right)}\\ \frac{1}{n_{c}n_{\mathrm{cc}}},\;\;\{\begin{array}{l} x_{j}\in L^{\left(c\right)},x_{i}\in L^{\left(\mathrm{cc}\right)}\\ x_{i}\in L^{\left(c\right)},x_{j}\in L^{\left(\mathrm{cc}\right)} \end{array}\\ 0 \end{array}$$

### 2.3 Induction Learning

The DPC criterion effectively addresses the challenges of domain distribution alignment and noise interference. Building upon this, we dedicate to achieving two core objectives in the process of target domain knowledge inference: (1) maintaining the consistency of geometric structures between the source domain and the target domain, ensuring that label information for neighboring samples remains consistent; and (2) striving simultaneously to minimize the structural risk loss between the source domain and the target domain. Through the description of the target task, the general form of the target risk function can be described in Equation (12):


(12) $$R\left(f_{W}\left(P\left(X\right)\right),Y\right)=\Omega_{Y}+\Omega_{W}$$

where $\Omega_{Y}$ represents the joint knowledge transfer and label propagation loss, which preserves the geometric structure consistency of the sample in both the source and target domains, and $\Omega_{W}$ includes the structural risk loss terms of the source domain and target domain. Next, we will design these two terms separately.

#### 2.3.1 Label Propagation

Firstly, we define the undirected weighted graph on the entire domain as G=X,M , and let $M\in {\mathbb{R}}^{N\times N}$ be the weight matrix and $M_{ij}=M_{ji}\geq 0$ . The calculation method for $M_{ij}$ is defined in Equation (13):


(13) $$M_{ij}=\left\{\begin{array}{l} \exp\left(-\frac{{\left\|x_{i}-x_{j}\right\|}^{2}}{2\sigma^{2}}\right),x_{i}\in Ne\left(x_{j}\right)\;\mathrm{or}\;x_{j}\in Ne\left(x_{i}\right)\\ 0,\mathrm{otherwise} \end{array}\right.$$

where $x_{k}\in Ne\left(x_{m}\right)$ indicates that $x_{k}$ is a neighbor of $x_{m}$ , σ controls the local influence range of the Gaussian kernel function and is also a hyper-parameter. The larger σ , the greater local influence range. Conversely, the smaller local influence range. When σ is fixed, the value of $M_{ij}$ decreases monotonically as the distance between $x_{i}$ and $x_{j}$ increases.

By combining source domain knowledge transfer and the graph Laplacian matrix (Long et al., 2013; Wang et al., 2018), label propagation modeling is performed as:


(14) $$\Omega_{Y}=\min_{Y}tr\left(Y^{T}LY\right)$$

where $Y=\left[Y_{s};Y_{t}\right]\in {\mathbb{R}}^{N\times C}$ , $Y_{t}$ is the label matrix of the target domain. If a sample in the target domain is unlabeled, the corresponding label value in $Y_{t}$ is all zeros. $Y_{s}$ is the label matrix of the source domain. $L=M-D\in {\mathbb{R}}^{N\times N}$ is the Laplacian graph matrix (Long et al., 2013) and D is a diagonal matrix with $D_{ii}=\overset{N}{\underset{j=1}{\sum }}M_{ij}$ .

#### 2.3.2 Design of Structural Risk

In our method, the source domain classifier $f_{s}$ and target domain classifier $f_{t}$ are defined as $f_{s}={\left(W_{s}^{'}\right)}^{T}X^{s}+b_{s}$ and $f_{t}={W_{t}^{'}}^{T}X^{t}+b_{t}$ , where $b_{s}$ ( $b_{t}$ ) is the bias term for the source domain (target domain), and $W_{s}^{o}$ ( $W_{t}^{'}$ ) is the parameter for the source domain (target domain). Let $\tilde{W}s=\left[W_{s}^{o},b_{s}\right]$ , ${\tilde{X}}^{s}=\left[X^{s},1\right]$ , ${\tilde{W}}_{t}=\left[W_{t}^{o},b_{t}\right]$ , and ${\tilde{X}}^{t}=\left[X^{t},1\right]$ . Then, the two classifiers can be rewritten as ${\tilde{f}}_{s}={{\tilde{W}}_{s}}^{T}{\tilde{X}}^{s}$ and ${\tilde{f}}_{t}={{\tilde{W}}_{t}}^{T}{\tilde{X}}^{t}$ . Let $W=\left[{\tilde{W}}_{s},{\tilde{W}}_{t}\right]$ and $\tilde{X}=\left[{\tilde{X}}^{s},{\tilde{X}}^{t}\right]$ . By combining the two classifiers into a single classifier, we get: $F\left(W\right)={\hat{X}}^{T}W$ .

According to the least squares loss function, the problem of minimizing structural risk for both domains can be described as:


(15) $$\Omega_{W}=\overset{C}{\underset{c=0}{\sum }}\overset{N}{\underset{k=1}{\sum }}\lambda_{k,c}^{2}{\left\|{x_{k,c}}^{T}W-y_{k}\right\|}_{H}^{2}+\rho {\left\|W\right\|}_{2,1}$$

where the first term is the structural risk loss term with $y_{k}\in Y$ . The second term is the constraint of the classification model. The features can be selected by applying $l_{2,1}$ regularization. It can effectively control the model complexity to a certain extent for preventing the target classification model from over-fitting.

Since the classification task ensures the reliability of predictions through the dual prediction of the label matrix Y and the decision function W , combining Equations 14 and 15 constitutes the target classification function. We can describe it as following:


(16) $$\begin{array}{l} R\left(f_{W}\left(P\left(X\right),Y\right)\right)=tr\left(Y^{T}LY\right)+\\ \overset{C}{\underset{c=0}{\sum }}\overset{N}{\underset{k=1}{\sum }}\lambda_{k,c}^{2}{\left\|{x_{k,c}}^{T}W-y_{k}\right\|}_{H}^{2}+\rho {\left\|W\right\|}_{2,1}\\ s.t.,0\leq \lambda_{k,c}\leq 1,YY^{T}=I \end{array}$$

### 2.4 Final formulation

Combining the intra-class attraction term in Equation 10, the inter-class repulsion term in Equation 11, and the induction learning in Equation 16, the final optimization formulation of the EDPC method can be described as following:


(17) $$\begin{array}{l} \Theta \left(\lambda ,P,Y^{t},W\right)\\ =\min_{\lambda_{k,c},Y,W}\sum_{c=0}^{C}\sum_{k=1}^{N}\lambda_{k,c}^{2}{\left\|P^{T}{x_{k,c}}^{T}W-y_{k}\right\|}_{H}^{2}+\alpha tr\left(Y^{T}LY\right)\\ \;+\rho {\left\|W\right\|}_{2,1}+\sum_{c=0}^{C}\sum_{k=1}^{N}\lambda_{k,c}^{2}{\left\|P^{T}x_{k,c}-P^{T}\mu_{c}\right\|}_{H}^{2}\\ \;+\theta \sum_{c=0}^{C}\sum_{k=1}^{N}\left(\lambda_{k,c}^{2}\ln\lambda_{k,c}^{2}-\lambda_{k,c}^{2}\right)\\ \;-\beta {\sum_{c=1}^{C}\left\|\frac{1}{nc}\sum_{x_{i}\in L^{\left(c\right)}}P^{T}x_{i}-\frac{1}{\sum_{\mathrm{cc}}n_{\mathrm{cc}}}\sum_{x_{j}\in L^{\left(\mathrm{cc}\right)}}P^{T}x_{j}\right\|}^{2}\\ s.t.,0\leq \lambda_{k,c}\leq 1,YY^{T}=I,\;P^{T}P=I \end{array}$$

where ρ , α , θ , and β are the balance parameters.

Once all model parameters are obtained, knowledge inference in the target domain can be achieved. By maximizing the use of discriminative information from the source domain, the two classifiers ${\tilde{f}}_{s}$ and ${\tilde{f}}_{t}$ are linearly combined, and this linear fusion model is used for target domain knowledge inference. The fusion form can be written as follows:


$$j=\arg\max_{j}{\left(y_{i}^{t}=\upsilon {\tilde{f}}_{s}\left(x_{i}^{t}\right)+\left(1-\upsilon \right){\tilde{f}}_{t}\left(x_{i}^{t}\right)\right)}_{j}\text{,}$$

where $\upsilon \in \left[0,1\right]$ is an adjustable parameter that balances the two classifiers. To emphasize the importance of discriminative information from the source domain as prior knowledge, we let υ=0.9 based on experience.

## 3 Optimization

The optimization problem of EDPC is a non-convex problem with respect to $\lambda_{k,c}$ , P , W , and Y This paper adopts an alternating iterative optimization strategy to solve these parameters, ensuring that each optimization variable has a closed-form analytical solution.

### 3.1 Update $\lambda_{k,c}$ as given W , P , and Y

Since the third and fifth terms in Equation 17 do not involve the calculation of $\lambda_{k,c}$ , the optimization solution of EDPC is described as follows:


(18) $$\begin{array}{l} \min_{\lambda_{k,c}}P_{1}=\min\sum_{c=0}^{C}\sum_{k=1}^{N}\lambda_{k,c}^{2}{\left\|P^{T}x_{k,c}-P^{T}\mu_{c}\right\|}_{H}^{2}\\ \;-\theta \sum_{c=0}^{C}\sum_{k=1}^{N}(-\lambda_{k,c}^{2}\ln\lambda_{k,c}^{2}+\lambda_{k,c}^{2})+\\ \;+\sum_{c=0}^{C}\sum_{k=1}^{N}\lambda_{k,c}^{2}{\left\|P^{T}{x_{k,c}}^{T}W-y_{i}\right\|}_{H}^{2}\\ s.t.0\leq \lambda_{k,c}\leq 1 \end{array}$$

**Theorem 3.** The optimal solution to the original optimization problem of the objective function (18) is:


(19) $$\lambda_{k,c}=\exp\left(-\frac{J}{\theta }\right)$$

where,


$$\begin{array}{l} J=\overset{C}{\underset{c=0}{\sum }}\overset{N}{\underset{k=1}{\sum }}{\left\|P^{T}{x_{k,c}}^{T}W-y_{k}\right\|}_{H}^{2}\\ +\overset{C}{\underset{c=0}{\sum }}\overset{N}{\underset{k=1}{\sum }}{\left\|P^{T}x_{k,c}-P^{T}\mu_{c}\right\|}_{H}^{2}\text{.} \end{array}$$

**Proof:** Taking the partial derivative of Equation 18 with respect to the variable $\lambda_{k,c}$ and setting it to zero, we get:


(20) ∂P1∂λk,c=2∑c=0C∑k=1Nλk,cPTxk,c−PTμcH2+2θ∑c=0C∑k=1Nλk,clnλk,c2+2∑c=0C∑k=1Nλk,cPTxk,cTW−YH2=0

By combining like terms and rearranging Equation 20, the solution for $\lambda_{k,c}$ can be obtained as Equation 19, thus proving the theorem.

According to Theorem 3, the matching contribution of any sample can be derived from Equation 19.

### 3.2 Update W as given Y , P , and $\lambda_{k,c}$

Since the first to third terms in Equation 17 do not involve the calculation of W , the optimization solution formula for EDPC is described as follows:


(21) $$\begin{array}{l} P_{2}=\min_{W}\overset{C}{\underset{c=0}{\sum }}\overset{N}{\underset{k=1}{\sum }}\lambda_{k,c}^{2}{\left\|P^{T}{x_{k,c}}^{T}W-y_{k}\right\|}_{H}^{2}+\rho {\left\|W\right\|}_{2,1}\\ =\min_{W}\lambda {\left\|P^{T}X^{T}W-Y\right\|}_{H}^{2}+\rho {\left\|W\right\|}_{2,1} \end{array}$$

where, $\lambda \in {\mathbb{R}}^{N\times C}$ and each element is $\lambda_{k,c}^{2}$ . $\lambda_{k,c}$ represents the membership value of $x_{k}$ belonging to the *c*-*th* category.

**Theorem 4.** The optimal solution to the original optimization problem of the objective Function (21) is:


(22) W=AY

where $A={\left(\lambda P^{T}XX^{T}P+\rho U\right)}^{-1}PX$ .

**Proof:** According to Equation 19, let ∂P2∂W=0 and solve for W , that is


(23) ∂P2∂W=2λXXTW−Y+2ρUW=0

where, $\frac{\partial \rho {\left\|W\right\|}_{2,1}}{\partial W}=UW$ , where U is a diagonal matrix with diagonal elements $U_{ii}=\frac{1}{\left\|w_{i}\right\|},\left(i=1,\ldots ,d\right)$ , and $w_{i}$ is the *i-th* row vector of matrix W . By rearranging Equation 23, the analytical solution for W can be derived as given in Equation 22.

### 3.3 Update Y by fixing W , P , and $\lambda_{k,c}$

Since the first, second, and fifth terms in Equation 17 do not involve the calculation of Y , and substituting the result W=AY into Equation 17, and the constraint ${YY}^{T}=I$ can reduce the interference information in the obtained label matrix Y , the objective form for optimizing and solving Y is represented as:


(24) $$\begin{array}{l} P_{3}=\min_{Y^{T}Y=I}\alpha tr\left(Y^{T}LY\right)\\ +\overset{C}{\underset{c=0}{\sum }}\overset{N}{\underset{k=1}{\sum }}\lambda_{k,c}^{2}{\left\|P^{T}x_{k}^{T}W-y_{k}\right\|}_{H}^{2}\\ =\min_{Y^{T}Y=I}\alpha tr\left(Y^{T}LY\right)\\ +\lambda {\left\|P^{T}X^{T}AY-Y\right\|}_{H}^{2}\\ =\min_{Y^{T}Y=I}tr\left(Y^{T}HY\right) \end{array}$$

where, $H=\alpha L+\lambda B^{T}B$ , $B=P^{T}X^{T}A-I$ . The optimization Problem (24) is a standard singular value decomposition problem. Y consists of the eigenvectors of matrix H , so the optimal solution for Y can be obtained by solving the singular value decomposition of matrix H .

### 3.4 Update P by fixing W , Y , and $\lambda_{k,c}$

From Equation 17, it can be seen that the optimization solution for EDPC can be described as:


(25) $$\begin{array}{l} P_{4}=\min_{P^{T}P=I}\sum_{c=0}^{C}\sum_{k=1}^{N}\lambda_{k,c}^{2}{\left\|P^{T}{x_{k,c}}^{T}W-y_{k}\right\|}_{H}^{2}\\ \;+\sum_{c=0}^{C}\sum_{k=1}^{N}\lambda_{k,c}^{2}{\left\|P^{T}x_{k,c}-P^{T}\mu_{c}\right\|}_{H}^{2}\\ \;-\beta {\sum_{c=1}^{C}\left\|\frac{1}{nc}\sum_{x_{i}\in L^{\left(c\right)}}P^{T}x_{i}-\frac{1}{\sum_{\mathrm{cc}}n_{\mathrm{cc}}}\sum_{x_{j}\in L^{\left(\mathrm{cc}\right)}}P^{T}x_{j}\right\|}^{2} \end{array}$$

**Theorem 5.** The optimal solution to the original optimization problem of the objective Function (25) is:


(26) $$P=\frac{1}{2}\overset{C}{\underset{c=0}{\sum }}\overset{N}{\underset{k=1}{\sum }}\lambda_{k,c}^{2}x_{k}Wy_{k}O^{-1}$$

where ϖ is the balancing parameter of the constraint term $P^{T}P=I$ .


$$\begin{array}{l} O=\overset{C}{\underset{c=0}{\sum }}\overset{N}{\underset{k=1}{\sum }}\lambda_{k,c}^{2}{\left\|x_{k,c}-\mu_{c}\right\|}_{H}^{2}-\beta XM_{\mathrm{cc}}X^{T}\\ \;+\varpi +\overset{C}{\underset{c=0}{\sum }}\overset{N}{\underset{k=1}{\sum }}\lambda_{k,c}^{2}x_{k}x_{k}^{T}WW^{T} \end{array}$$

**Proof:** According to Equation 25, let ∂P4∂P=0 , solve for P , it is shown in Equation (27):


(27) ∂P4∂P=P∑c=0C∑k=1Nλk,c2xk,c−μcH2−βPXMccXT+ϖP+PWWT∑c=0C∑k=1Nλk,c2xkxkT−12W∑c=0C∑k=1Nλk,c2xkxkTyk=0

The solution for P can be obtained as Equation 26, thus Theorem 5 is proved.

## 4 Algorithm

### 4.1 Algorithm description

In the context of unsupervised domain adaptation where the target domain lacks labeled data, achieving semantic alignment between domains relies on initial label information from the target domain. The initial label information of the target domain samples can be obtained through the following three strategies (Liang et al., 2019): (1) by a random method; (2) by setting all labels to zero; (3) by performing clustering on the target domain data using a model trained from the source domain data. Strategies (1) and (2) are cold start methods. Strategy (3) is a warm start method, which is usually more beneficial for the subsequent learning process. Therefore, we choose the third strategy to initialize the prior information of the target domain, thereby initializing $\lambda_{k,c}$ , W , P , and Y . EDPC utilizes an iterative optimization strategy, which is a common approach in multi-objective optimization. The iterative process of the algorithm will stop when the following conditions are met: $\left|\Theta \left(\lambda_{k,c}^{z},P^{z},W^{z},Y^{z}\right)-\Theta \left(\lambda_{k,c}^{z-1},P^{z-1},W^{z-1},Y^{z-1}\right)\right|<\varepsilon$ , where $\Theta \left(\lambda_{k,c}^{z},P^{z},W^{z},Y^{z}\right)$ represents the value of the objective function at the *z-th* iteration, and ε is a predefined threshold. The complete learning process of the proposed method is given in Algorithm 1.

#### ALGORITHM 1 : Domain adaptation learning based on EDPC

**Input:** The source domain data $\left\{{\hat{X}}_{s},Y_{s}\right\}$ , the target domain data ${\hat{X}}_{t}$ , unknown target domain labels $Y_{t}$ (initialized via clustering), and parameters ρ,α,ϑ,β,ϖ , the threshold for iteration termination ε , and the maximum number of iterations Z .

**Output:** The contribution matrix λ , which represents the matching contributions of each instance at the mean points of various categories in the overall domain; the shared subspace P ; the decision function W on the source and target domain datasets; and the label matrix Y .

**Procedure:**

1. Initialize the label values for the unlabeled data in the target domain.
2. Calculate the mean values $\mu_{t,c}$ and $\mu_{s,c}$ for different categories in the target domain and source domain, respectively, where c=0,1,2,…,C .
3. Then calculate the mean values $\mu_{c}$ for different categories in the overall domain as $\mu_{c}=\frac{1}{2}\left(\mu_{s,c}+\mu_{t,c}\right)$ .
4. Obtain the initial values $\lambda_{k,c}^{0}$ for $\lambda_{k,c}$ using Equation 19.
5. Obtain the initial values $W^{0}$ for W using Equation 22.
6. Obtain the initial values $Y^{0}$ for Y using Equation 24.
7. Obtain the initial values $P^{0}$ for P using Equation 26.
8. Compute the value of the objective function $\Theta \left(\lambda_{k,c}^{0},P^{0},W^{0},Y^{0}\right)$ .
9. Repeat the following steps sequentially from z=0toZ :

{

1. Update the values of $\lambda_{k,c}$ to $\lambda_{k,c}^{z}$ using Equation 19.
2. Obtain the updated values $W^{z}$ for W using Equation 22.
3. Obtain the updated values $Y^{z}$ for Y using Equation 24.
4. Obtain the updated values *P*^2^ for P using Equation 26.
5. Update the value of the objective function to $\Theta \left(\lambda_{k,c}^{0},P^{0},W^{0},Y^{0}\right)$ .
6. If $|\Theta \left(\lambda_{k,c}^{z},P^{z},W^{z},Y^{z}\right)-\Theta \left(\lambda_{k,c}^{z-1},P^{z-1},W^{z-1},Y^{z-1}\right)|<\varepsilon$ , terminate the repetition and return the matrices λ , P , W , and Y ; otherwise, go back to step 9.1 and continue the calculation until the condition in step 9.5 is satisfied.

}

### 4.2 Generalization Analysis

Rademacher complexity is an effective measure of a function set’s capacity to fit noise (Ghifary et al., 2017; Tao and Dan, 2021). Therefore, we will derive the generalization error bound for the proposed method using Rademacher complexity. Let $H:=\left\{X\to Y\right\}$ be a set of hypothesis functions in the RKHS H space, where X is a compact set and Y is a label space. Given a loss function $\mathrm{loss}\left(\cdot ,\cdot \right):Y\times Y\to {\mathbb{R}}_{+}$ and a neighborhood distribution D on X , the expected loss between two hypothesis functions $h,\tilde{h}\in H$ is defined as:


$$L_{D}\left(h,\tilde{h}\right)=E_{x∼D}\left[\mathrm{loss}\left(h\left(x\right),\tilde{h}\left(x\right)\right)\right]$$

The difference in domain distributions between the source domain distribution ℙ and the target domain distribution ℚ can be defined in Equation (29):


(29) $$\mathrm{disc}\left(\mathbb{P},\mathbb{Q}\right)=\underset{h,\tilde{h}\in H}{\sup}\left\{L_{\mathbb{P}}\left(h,\tilde{h}\right)-L_{\mathbb{Q}}\left(h,\tilde{h}\right)\right\}$$

Let $f_{\mathbb{P}}$ and $f_{\mathbb{Q}}$ be the true label functions for ℙ and ℚ , respectively, and let the corresponding optimized hypothesis functions be:


$$h_{\mathbb{P}}^{*}:=\underset{h\in H}{\mathrm{argmin}}L_{\mathbb{P}}\left(h,f_{\mathbb{P}}\right)$$

$$h_{\mathbb{Q}}^{*}:=\underset{h\in H}{\mathrm{argmin}}L_{\mathbb{Q}}\left(h,f_{\mathbb{Q}}\right)$$

Their corresponding expected loss is denoted as $L_{\mathbb{P}}\left(h_{\mathbb{Q}}^{*},h_{\mathbb{P}}^{*}\right)$ . Our EDPC method aims to achieve the empirical loss target $L_{\mathbb{P}}\left(h_{\mathbb{Q}}^{*},h_{\mathbb{P}}^{*}\right)$ through the objective function $R\left(Y,W\right)$ .

The following theorem provides the generalization error bound for the proposed method:

Theorem 6 (Generalization Error Bound; Nie et al., 2010). Let $H:=\left\{f\in H:X\to \mathbb{R},{\left\|f\right\|}_{H}\leq 1\;\mathrm{and}\;{\left\|f\right\|}_{\infty }\leq r\right\}$ be a function set in the reproducing kernel Hilbert space (RKHS) H . Consider $X_{X}^{\mathbb{P}}=\left(x_{1}^{s},\ldots ,x_{n_{s}}^{s}\right)∼\mathbb{P}$ and $X_{X}^{\mathbb{Q}}=\left(x_{1}^{t},\ldots ,x_{n_{t}}^{t}\right)∼\mathbb{Q}$ as datasets from the source domain and the target domain, respectively. Assume the loss function loss (.) is q-Lipschitz , mapping $\mathrm{loss}\left(\cdot ,\cdot \right):Y\times Y\to \left[0,q\right]$ . For a,b∈Y×Y , the condition $\left|\mathrm{loss}\left(a\right)-\mathrm{loss}\left(b\right)|=q|a-b\right|$ holds. The generalization error bound for any hypothesis function h∈H , with a probability of at least 1−δ , having Rademacher complexity ${ℜ}_{X_{X}^{\mathbb{P}}}\left(H\right)$ on $X_{X}^{\mathbb{P}}$ , is shown in Equation (30):


(30) $$\begin{array}{l} L_{\mathbb{Q}}\left(h,f_{\mathbb{Q}}\right)-L_{\mathbb{Q}}\left(h_{\mathbb{Q}}^{*},f_{\mathbb{Q}}\right)\leq L_{\mathbb{P}}\left(h,h_{\mathbb{P}}^{*}\right)+2q{ℜ}_{X_{X}^{\mathbb{P}}}\left(H\right)\\ +3q\sqrt{\frac{\log\frac{2}{\delta }}{2N}}+8q\sqrt{\Omega \left(P\left(X_{s}\right),P\left(X_{t}\right)\right)}+R\left(W,Y\right) \end{array}$$

where ${ℜ}_{X_{X}^{\mathbb{P}}}\left(H\right)$ is the Rademacher complexity, $\Omega \left(P\left(X^{s}\right),P\left(X^{t}\right)\right)$ is the disctriminative possibilistic clustering distribution distance measure and is composed by possibilistic clustering distribution distance measure with intra-class alignment item $\Omega_{\lambda }\left(P\left(X^{s}\right),P\left(X^{t}\right)\right)$ and disctriminative item $\Omega_{\mathrm{rep}}\left(P\left(X^{s}\right),P\left(X^{t}\right)\right)$ (i.e., $\Omega \left(P\left(X^{s}\right),P\left(X^{t}\right)\right)=\alpha \Omega_{\lambda }\left(P\left(X^{s}\right),P\left(X^{t}\right)\right)-\beta \Omega_{\mathrm{rep}}\left(P\left(X^{s}\right),P\left(X^{t}\right)\right)$ ).

Theorem 6 demonstrates that the disctriminative possibilistic clustering distribution distance measure $\Omega \left(P\left(X^{s}\right),P\left(X^{t}\right)\right)$ and the model alignment function $R\left(W,Y\right)$ can jointly control the generalization error bound of the proposed method. Consequently, by minimizing both the disctriminative possibilistic distribution distance between domains and the model bias, the proposed method can effectively enhance its generalization performance in domain adaptation. Experimental results on real-world datasets also support this conclusion.

## 5 Experiments

To validate the effectiveness of the proposed EDPC in the cross-domain emotion recognition, this section systematically compares and analyzes the performance of the EDPC method with current state-of-the-art unsupervised domain adaptation techniques on several key EEG datasets (i.e., SEED and SEED-IV).

### 5.1 Databases description

To ensure a fair comparison with state-of-the-art (SOTA) methods, extensive experiments were conducted for effective validation using two well-known open datasets: SEED (Zheng and Lu, 2015a) and SEED-IV (Zheng et al., 2018). The SEED datasets comprises data collected from 15 subjects, each participating in three sessions held at different times. Each session includes 15 trials, featuring three types of emotional stimuli: negative, neutral, and positive. Similarly, the SEED-IV datasets also involves 15 subjects, each undergoing three sessions at different times. Each session in SEED-IV consists of 24 trials, with four emotional stimuli: happy, sad, fearful, and peaceful.

EEG signals for both datasets (SEED and SEED-IV) were simultaneously recorded using a 62-channel ESI Neuroscan system. During EEG signal preprocessing, the data were down-sampled to a rate of 200 Hz. Environmental noise was manually removed, and the data were filtered using a 0.3 Hz-50 Hz band-pass filter. To make a fair comparison with the existing studies on the two benchmark databases, we also use the pre-computed differential entropy (DE) features as the model input. Specifically, for each trial, the EEG data was divided into a number of 1-s segments, and the DE features were extracted from each 1-s segment at the given five frequency bands [Delta (1–3 Hz), Theta (4–7 Hz), Alpha (8–13 Hz), Beta (14–30 Hz), and Gamma (31–50 Hz)] from the 62 channels. Then, for each 1-s segment, it was represented by a 310-dimensional feature vector (5 frequency bands × 62 channels), which was further filtered by a linear dynamic system method for smooth purpose (Shi and Lu, 2010).

### 5.2 Experiment Settings

Before delving into the detailed analysis of the experimental results, it is essential to fine-tune the hyperparameters in the EDPC strategy. Empirical evidence suggests that the hyperparameters θ and α serve to balance the trade-off between fuzziness and local structure preservation in the objective Function (17) for both the source and target domains. Meanwhile, the other two hyperparameters β and ρ can still be adjusted to, respectively, balance the influence of class exclusion and feature selection. Therefore, these two parameters play a crucial role in the final performance of the algorithm.

Given that parameter setting remains a challenging issue in the field of machine learning, this study adopts an experience-based parameter space exploration strategy to determine the optimal parameter configuration. This, in turn, allows for the evaluation of various methods on the datasets and the recording of the best performance for each method. Except in special cases, all related methods will undergo fine-tuning to achieve optimal results. Regarding the potential geometric structure of the data, it is associated with the neighborhood size chosen when constructing the Laplacian matrix. Experimental observations show that the model’s performance is slightly sensitive to changes in neighborhood size when the neighborhood is small. Therefore, when constructing the nearest neighbor graph in EDPC, this study uses grid search within the range $\left\{3,5,10,15,17\right\}$ to determine the optimal number of neighbors and reports the highest recognition accuracy results obtained under this optimal parameter configuration.

Additionally, for all methods implemented in this paper, a Gaussian kernel $K\left(x,x_{i}\right)=\exp\left(-x-{x_{i}}^{2}/2\sigma^{2}\right)$ is used in both the source and target domains, where σ is determined by minimizing the Maximum Mean Discrepancy (MMD) to establish a benchmark. Based on prior experience, σ is initially set to the square root of the average norm of the binary training data, and for multiclass classification, σ is adjusted to $\sigma \sqrt{C}$ (where C represents the number of classes). The underlying geometric structure relies on *k* neighbors for computing the Laplacian matrix. In our experiments, we observed that performance slightly varies when *k* is not large. Consequently, to construct the nearest neighbor graph in EDPC, we conduct a grid search to determine the optimal number of nearest *k* neighbors from the set $\left\{3,5,10,15,17\right\}$ , and report the best recognition accuracy from the optimal parameter configuration.

Before presenting the detailed evaluation, it is essential to explain the tuning process for the hyper-parameters of EDPC. Based on prior experience, the parameter β is used to balance the fuzzy entropy and the alignment of domain probability distributions in the objective Function (16). The parameters α and ρ are adjustable and used to balance the importance of structure description and feature selection. Given that parameter uncertainty remains an open issue in machine learning, we rely on previous work experience to determine these parameters. Consequently, we evaluate all methods on the datasets by empirically searching the parameter space to identify the optimal settings and report the best results for each method. Except for special cases, all parameters of relevant methods are fine-tuned to achieve optimal results.

Since unsupervised domain adaptation lacks target labels for standard cross-validation, we employ a leave-one-subject-out strategy on the SEED and SEED-IV datasets (detailed in Section 6.2). We identify the optimal parameter values from the set { $10^{-6}$ , $10^{-5}$ , … $10^{5}$ , $10^{6}$ } by achieving the highest average accuracy on these datasets using the aforementioned method. This strategy typically constructs an effective EDPC model for unsupervised domain adaptation, and a similar approach is used to find optimal parameter values for other domain adaptation methods.In the subsequent sub-sections, a series of experiments is designed to test the sensitivity of the proposed EDPC method to parameter selection (see Section 6.4.1), verifying that EDPC can maintain stable performance across a wide range of parameter values. Additionally, the hyper-parameters for other methods are selected according to their original literature.

### 5.3 Experimental protocols

To fully verify the robustness and stability of the proposed method, this paper employs three different validation protocols (leave-one-subject-out; Zhang et al., 2020) to compare the proposed method with the latest methods.

1. Single-subject cross-session leave-one-session-out cross-validation. In line with existing methods, a time series cross-validation approach is utilized here, where past data is leveraged to predict current or future data. For each subject, the first two sessions are designated as the source domain, while the latter session serves as the target domain. The final results are determined by calculating the average accuracy and standard deviation across all subjects.
2. Cross-subject single-session leave-one-subject-out cross-validation. This validation scheme is the most widely used in emotion recognition tasks based on EEG data (Li J. et al., 2020; Luo et al., 2018). In this approach, one session’s data from a subject is treated as the target domain, while the data from the remaining subjects serve as the source domain. The training and validation process is repeated until each subject has been used as the target once. Consistent with other studies, we only consider the first session for this type of cross-validation.
3. Cross-subject cross-session leave-one-subject-out cross-validation. To comprehensively assess the robustness of the model on unseen subjects and trials, this paper employs a rigorous leave-one-out cross-subject cross-session method for evaluation. In this approach, all session data from a single subject are designated as the target domain, while data from all sessions of the remaining subjects serve as the source domain. The training and validation process is repeated until each subject’s sessions have been used as the target domain once. Given the variations between subjects and sessions, this evaluation protocol presents a substantial challenge to the effectiveness of models in EEG-based emotion recognition tasks.

### 5.4 Experimental results

Specifically, in the following tables of experimental results, the bold values in each table are the best accuracy performance results achieved by the compared methods. Pacc denotes the average accuracy performance of each method. In following tables, EDPC denotes the method proposed by us. During the implementation of the experiments, the features of the data were initially extracted using shallow technical means. When labeled as EDPC+ResNet101, it indicates that we employed the deep neural network ResNet101 for data feature extraction in the experimental process.

#### 5.4.1 Single-subject cross-session

We calculate the average and standard deviation of each subject’s experimental results, the cross-session validation results for each subject on different datasets (i.e., SEED and SEED-IV) show in Tables 1, 2, respectively. When the proposed EDPC method is compared with the traditional machine learning methods on both SEED and SEED-IV, the EDPC obtained the best accuracy performance, even DICE. It indicates that the EDPC method with discrimination has a more significant noise suppression effect.

**Table 1:** The mean accuracies (%) and standard deviations (%) of emotion recognition on SEED database using single-subject cross-session leave-one-subject-out cross-validation.

Methods	Pacc	Methods	Pacc
Traditional machine learning methods			
RF (Breiman, 2001)	76.42 ± 11.15	KNN* (Coomans and Massart, 1982)	72.96 ± 12.10
TCA* (Pan et al., 2011)	77.63 ± 11.49	CORAL (Sun et al., 2016)	84.18 ± 9.81
SA* (Li Y. et al., 2020)	67.79 ± 7.43	GFK* (Gong et al., 2012)	79.28 ± 7.44
DICE (Liang et al., 2019)	81.58 ± 7.55	EDPC	**82.31 ± 6.44**
MDDD (Luo et al., 2024)	81.27 ± 5. 47		
Deep learning methods			
DAN (Long et al., 2015)	89.16 ± 7.90	SimNet (Pinheiro, 2018)	86.88 ± 7.83
DDC (Tzeng et al., 2014)	91.14 ± 5.61	ADA (Li J. et al., 2020)	89.13 ± 7.13
DANN (Ganin et al., 2016)	89.45 ± 6.74	MMD (Li J. et al., 2020)	84.38 ± 12.05
JDA-Net (Li J. et al., 2020)	91.17 ± 8.11	DCORAL (Sun et al., 2016)	88.67 ± 6.25
PR-PL (Zhou et al., 2022)	93.18 ± 6.55	PARSE (Zhang and Etemad, 2022)	89.85 ± 5.06
EEGMatch (Zhou et al., 2023)	**94.70 ± 4.10**	EDPC+ResNet101	93.76 ± 5.82

Here, the model results reproduced by us are indicated by ‘*’. The bold value in table corresponds to the method with the best accuracy performance result among all the compared methods.

**Table 2:** The mean accuracies (%) and standard deviations (%) of emotion recognition on SEED-IV database using single-subject cross-session leave-one-subject-out cross-validation.

Methods	Pacc	Methods	Pacc
Traditional machine learning methods			
RF (Breiman, 2001)	60.27 ± 16.36	KNN (Coomans and Massart, 1982)	54.18 ± 16.28
TCA* (Pan et al., 2011)	59.49 ± 12.07	CORAL* (Sun et al., 2016)	66.88 ± 14.67
SA* (Li Y. et al., 2020)	56.94 ± 11.45	GFK* (Gong et al., 2012)	60.66 ± 10.00
DICE (Liang et al., 2019)	69.68 ± 12.52	EDPC	**71.39 ± 7.22**
MDDD (Luo et al., 2024)	68.81 ± 9.25		
Deep learning methods			
DCORAL (Sun et al., 2016)	65.10 ± 13.20	DAN (Long et al., 2015)	60.20 ± 10.20
DDC (Tzeng et al., 2014)	68.80 ± 16.60	MEERNet (Chen et al., 2021)	72.10 ± 14.10
PR-PL (Zhou et al., 2022)	74.62 ± 14.15	PARSE (Zhang and Etemad, 2022)	70.24 ± 8.47
EEGMatch (Zhou et al., 2023)	72.91 ± 8.34	EDPC+ResNet101	**76.58 ± 10.29**

Here, the model results reproduced by us are indicated by ‘*’. The bold value in table corresponds to the method with the best accuracy performance result among all the compared methods.

Additionally, in the experiments on the SEED datasets, the results from deep learning methods show that the EEGMatch method achieved the best performance among the deep learning methods. This is possibly because the mixup technique provided richer and more effective data information, which aided the model training, although the increased data volume naturally led to higher computational costs. Nevertheless, the EDPC method still obtained comparable performance and ranked closely its behind. This demonstrates the advantage of the EDPC method in distinguishing the single subject across sessions.

We can observe from the Table 3 that the EDPC method achieved the best performance on SEED-IV datasets with four different emotions (SEED has three different emotions), no matter what in the traditional machine learning or the deep learning methods. It signs that the EDPC method with “sames attract and opposites repel” characteristics can get more accuracy performance on finer-grained emotion recognition. The EDPC has stronger scalability in more nuanced emotion recognition tasks.

**Table 3:** The mean accuracies (%) and standard deviations (%) of emotion recognition on SEED database using cross-subject single-session leave-one-subject-out cross-validation.

Methods	Pacc	Methods	Pacc
Traditional machine learning methods			
TKL (Li et al., 2018c)	63.54 ± 15.47	T-SVM* (Li et al., 2018c)	68.57 ± 9.54
TCA (Pan et al., 2011)	63.64 ± 14.88	TPT* (Suykens and Vandewalle, 1999)	73.86 ± 11.05
KPCA (Suykens and Vandewalle, 1999)	61.28 ± 14.62	GFK (Gong et al., 2012)	71.31 ± 14.09
SA* (Li Y. et al., 2020)	66.00 ± 10.89	DICA (Ma et al., 2019)	69.40 ± 07.80
DNN (Suykens and Vandewalle, 1999)	61.01 ± 12.38	SVM (Suykens and Vandewalle, 1999)	58.18 ± 13.85
DICE (Liang et al., 2019)	74.22 ± 7.33	EDPC	82.34 ± 7.52
MDDD (Luo et al., 2024)	**84.57 ± 9.49**		
Deep learning methods			
DGCNN (Song et al., 2018)	79.95 ± 9.02	DAN (Long et al., 2015)	83.81 ± 8.56
RGNN (Zhong et al., 2020)	85.30 ± 6.72	BiHDM (Li Y. et al., 2020)	85.40 ± 7.53
WGAN-GP (Luo et al., 2018)	87.10 ± 7.10	MMD (Li J. et al., 2020)	80.88 ± 10.10
ATDD-DANN (Du et al., 2020)	90.92 ± 1.05	JDA-Net (Li J. et al., 2020)	88.28 ± 11.44
R2G-STNN (Li et al., 2019)	84.16 ± 7.63	SimNet* (Pinheiro, 2018)	81.58 ± 5.11
BiDANN (Li et al., 2018c)	83.28 ± 9.60	DResNet (Ma et al., 2019)	85.30 ± 8.00
ADA (Li J. et al., 2020)	84.47 ± 10.65	DANN (Ganin et al., 2016)	81.65 ± 9.92
PR-PL (Zhou et al., 2022)	93.06 ± 5.12	PARSE (Zhang and Etemad, 2022)	82.11 ± 5.83
EEGMatch (Zhou et al., 2023)	92.45 ± 06.85	EDPC+ResNet101	**94.79 ± 4.28**

Here, the model results reproduced by us are indicated by ‘*’. The bold value in table corresponds to the method with the best accuracy performance result among all the compared methods.

#### 5.4.2 Cross-subject single-session

Table 3 presents the model results for the recognition task using the leave-one-subject-out method within a single session, comparing them with the performance of the latest methods in the literature. All results are presented as mean ± standard deviation. The MDDD method achieved the best performance, the possible reason is: a more balanced impact of noise may arise when the different subjects in the same session faced consistent consistent environment, the MDDD adopted ensemble learning approach can effectively handle this kind of data. The MDDD method needs higher computational costs since it requires training and combining multiple models.

However, the EDPC achieved the best accuracy (82.34) with a standard deviation of 7.52 except MDDD method. It still maintains a comparable performance advantage overall among traditional machine learning methods. The recognition performance of EDPC surpasses that of the DICE method, indicating that the EDPC method handles noise better than the DICE method. It shows that the clustering hypothesis with fuzzy entropy can overcome the influence of noise and outliers in unsupervised classification.

When we compared to the latest deep learning methods, particularly deep transfer learning networks based on DANN (e.g., ATDD-DANN, R2GSTNN, BiHDM, BiDANN, WGAN-GP, PR-PL, EEGMatch), the proposed EDPC method demonstrated the best performance from Table 3. It effectively addresses issues of individual differences and noisy labels in aBCI applications, indicating that the EDPC method has better generalization performance and discrimination across subjects within the same session.

#### 5.4.3 Cross-subject cross-session

To validate the efficiency and stability of the EDPC method under cross-subject and cross-session conditions, this study uses cross-subject cross-session leave-one-out cross-validation on the SEED and SEED-IV databases to verify the proposed EDPC method. As shown in Tables 4, 5, the proposed EDPC method achieves the best performance when compared to traditional machine learning methods and deep learning methods. Moreover, Table 3 obtained better performance than Table 5 since the cross-subject cross-session is more complicated. We easy to see that the performance of EDPC method is better than the MDDD method. All these results demonstrate that the proposed EDPC method has higher recognition accuracy and better generalization ability in the face of more complex individual and environmental differences, indicating better emotional validity.

**Table 4:** The mean accuracies (%) and standard deviations (%) of emotion recognition on SEED database using cross-subject cross-session leave-one-subject-out cross-validation.

Methods	Pacc	Methods	Pacc
Traditional machine learning methods			
RF (Breiman, 2001)	69.60 ± 7.64	KNN (Coomans and Massart, 1982)	60.66 ± 7.93
SVM* (Suykens and Vandewalle, 1999)	62.24 ± 5.48	Adaboost (Zhu et al., 2006)	71.87 ± 5.70
TCA* (Pan et al., 2011)	65.31 ± 6.04	CORAL (Sun et al., 2016)	69.22 ± 4.11
SA (Li Y. et al., 2020)	61.41 ± 9.75	GFK* (Gong et al., 2012)	67.36 ± 6.52
DICE* (Liang et al., 2019)	73.56 ± 4.23	EDPC	**76.82 ± 7.14**
MDDD (Luo et al., 2024)	76.60 ± 6.79		
Deep learning methods			
DCORAL* (Sun et al., 2016)	80.87 ± 6.04	DAN* (Long et al., 2015)	82.51 ± 3.71
DDC (Tzeng et al., 2014)	82.17 ± 4.96	DANN* (Ganin et al., 2016)	84.79 ± 6.44
PR-PL (Zhou et al., 2022)	85.56 ± 4.78	PARSE (Zhang and Etemad, 2022)	82.44 ± 5.00
EEGMatch (Zhou et al., 2023)	86.30 ± 5.04	EDPC+ResNet101	**87.42 ± 6.15**

The bold value in table corresponds to the method with the best accuracy performance result among all the compared methods.

**Table 5:** The mean accuracies (%) and standard deviations (%) of emotion recognition on SEED-IV database using cross-subject cross-session leave-one-subject-out cross-validation.

Methods	Pacc	Methods	Pacc
Traditional machine learning methods			
RF (Breiman, 2001)	50.98 ± 9.20	KNN (Coomans and Massart, 1982)	40.83 ± 7.28
SVM (Suykens and Vandewalle, 1999)	51.78 ± 12.85	Adaboost (Zhu et al., 2006)	53.44 ± 9.12
TCA (Pan et al., 2011)	56.56 ± 13.77	CORAL (Sun et al., 2016)	49.44 ± 9.09
SA (Li Y. et al., 2020)	64.44 ± 9.46	GFK (Gong et al., 2012)	45.89 ± 8.27
KPCA (Suykens and Vandewalle, 1999)	51.76 ± 12.89	DNN (Suykens and Vandewalle, 1999)	49.35 ± 9.74
DICE (Liang et al., 2019)	66.75 ± 7.25	EDPC	**67.88 ± 5.21**
MDDD (Luo et al., 2024)	64.90 ± 10.25		
Deep learning methods			
DGCNN (Song et al., 2018)	52.82 ± 9.23	DAN (Long et al., 2015)	58.87 ± 8.13
RGNN (Zhong et al., 2020)	73.84 ± 8.02	BiHDM (Li Y. et al., 2020)	69.03 ± 8.66
BiDANN (Li et al., 2018c)	65.59 ± 10.39	DANN (Ganin et al., 2016)	54.63 ± 8.03
PR-PL (Zhou et al., 2022)	74.92 ± 7.92	PARSE (Zhang and Etemad, 2022)	69.78 ± 8.22
EEGMatch (Zhou et al., 2023)	73.60 ± 7.53	EDPC+ResNet101	**76.11 ± 6.69**

The bold value in table corresponds to the method with the best accuracy performance result among all the compared methods.

## 6 Discussion

To comprehensively study the model’s performance, this section evaluates the effects of different settings in EDPC. Please note that all results presented in this section are based on the SEED datasets, using the cross-subject single-session cross-validation evaluation protocol. The bold values in Table 6 is the best accuracy performance result achieved of this method. It’s best to review in color mode.

**Table 6:** The ablation study of our proposed model.

	Pacc
Ablation setting	
target prior information (5 labeled samples per category)	95.98 ± 5.22
only preserving the local structures on the source	92.29 ± 5.67
only preserving the local structures on the target	92.83 ± 4.81
imposing $l_{2}$ -norm on W	92.13 ± 5.83
fixed pseudo-labeling	91.48 ± 5.33
dynamic pseudo-labeling	94.71 ± 4.15
multiple kernel leaning	95.66 ± 3.20
Hyper-parameter controlling strategy	
ρ = 0 (Ignore the regularization term of W )	92.79 ± 4.40
fixed ρ = 100 for W regularization	93.54 ± 5.16
β = 0 (Ignore the regularization term of discriminative)	90.56 ± 3.25
fixed β =100 forthe regularization term of discriminative	94.74 ± 4.53
The proposed model	
EDPC + ResNet101	**94.79 ± 4.28**

The bold value in table corresponds to the method with the best accuracy performance result among all the compared methods.

### 6.1 Ablation study

This ablation study systematically explores the effectiveness of different components in the proposed model and presents the corresponding contributions of each component to the overall performance of the model. As shown in Table 6, adding 5 labeled data points per category in the target domain achieves a recognition accuracy (95.98 ± 5.22) very close to the recognition accuracy of EDPC (unsupervised learning; 92.19 ± 4.70). This decline indicates that prior label information in the target domain significantly enhances model performance and highlights the great potential of transfer learning in aBCI applications. Moreover, simultaneously preserving the local structure of data in both the source and target domains helps to improve model performance; otherwise, the recognition accuracy significantly decreases (92.29 ± 5.67 and 92.83 ± 4.81, respectively). When the l21 norm in W is replaced with the l2 norm, the model’s recognition accuracy drops to 92.13 ± 5.83. This result demonstrates that using the l21 constraint achieves better sample selection and denoising effects.

For the pseudo-labeling method, when switching from a fixed mode to a linear dynamic update, the corresponding accuracy increases from 91.48 to 94.71. When using an adaptive pseudo-labeling method based on nonlinear dynamics, the accuracy further improves to 94.79. When employing multi-kernel learning, the accuracy further increases to 95.66. These results indicate that multi-kernel learning helps rationalize the importance of different kernels in various scenarios and enhances the model’s generalization ability.

Next, we analyze the impact of different hyperparameters on the overall performance of the model. According to the experimental results, it can be observed that when the dynamic learning rate ρ varies from 0 to 100, the model accuracy continuously improves from 92.79 to 93.54. This indicates that a dynamic learning rate ρ is superior to a fixed value in terms of recognition accuracy. Additionally, the results suggest that the value of β directly affects the importance of the discriminative term in the model. When the discriminative term is removed, the model accuracy drops to 90.56, whereas when β = 100, the model accuracy reaches 94.74, which is close to the performance of EDPC. This demonstrates that the discriminative term plays an indispensable role in the model.

**Figure 1:**
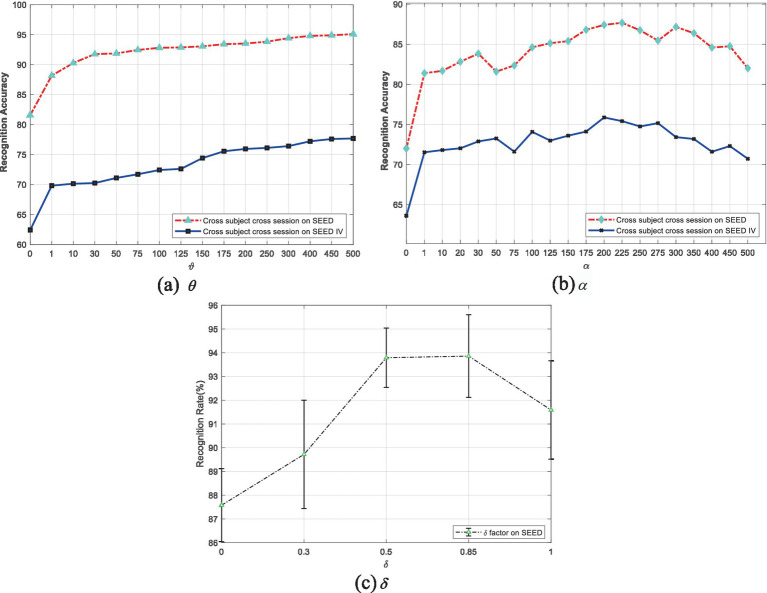
Performance variations of the EDPC method due to changes in parameters θ , α , and δ . **(a)** Adjusted for fuzzy entropy; **(b)** Adjusted for label propagation; **(c)** Weighted for the different classes centers of source domain.

Additionally, θ and θ are two balancing parameters used to optimize the performance of EDPC by weighting fuzzy entropy and local retention (referring to the source and target domains). As shown in Figure 1a, when θ is 0, the performance is poor. Performance jumps significantly when θ is 1, and it continues to improve steadily as θ increases. When θ reaches 400 or higher, the performance stabilizes. Therefore, based on the experimental results, setting θ around 400 can achieve the best EDPC performance.

From Figure 1b, it can be seen that when α is 0, the performance is poor. When α is 1, there is a performance jump. As α increases further, the performance improves slowly and becomes jittery. According to the trend observed in the experimental curve, α can be adjusted within the range of [100, 225] to achieve optimal EDPC performance.

Figure 1c shows that when δ is within the range of [0.5, 0.85], the performance is high, indicating that the source domain contains a significant amount of discriminative information, and the means of different categories play a more important role. When δ takes larger values up to 1 (completely removing the target domain’s mean information), the performance declines, further demonstrating that the mean information of the target domain also plays an auxiliary role.

### 6.2 Effect of noisy labels

To further validate the robustness of the model in the presence of noisy labels, we conducted an experiment where noise was randomly added to the source labels at different proportions, and the corresponding model performance on unseen target data was tested. Specifically, we replaced a certain proportion of the true labels with randomly generated labels and trained the model using semi-supervised learning. Then, the trained model’s performance was evaluated on the target domain. It is important to note that noise was only added to the source domain data, while the target domain was used for model evaluation. In the implementation, the noise proportions were adjusted to 5, 15, 25, and 30% of the source domain data.

**Figure 2:**
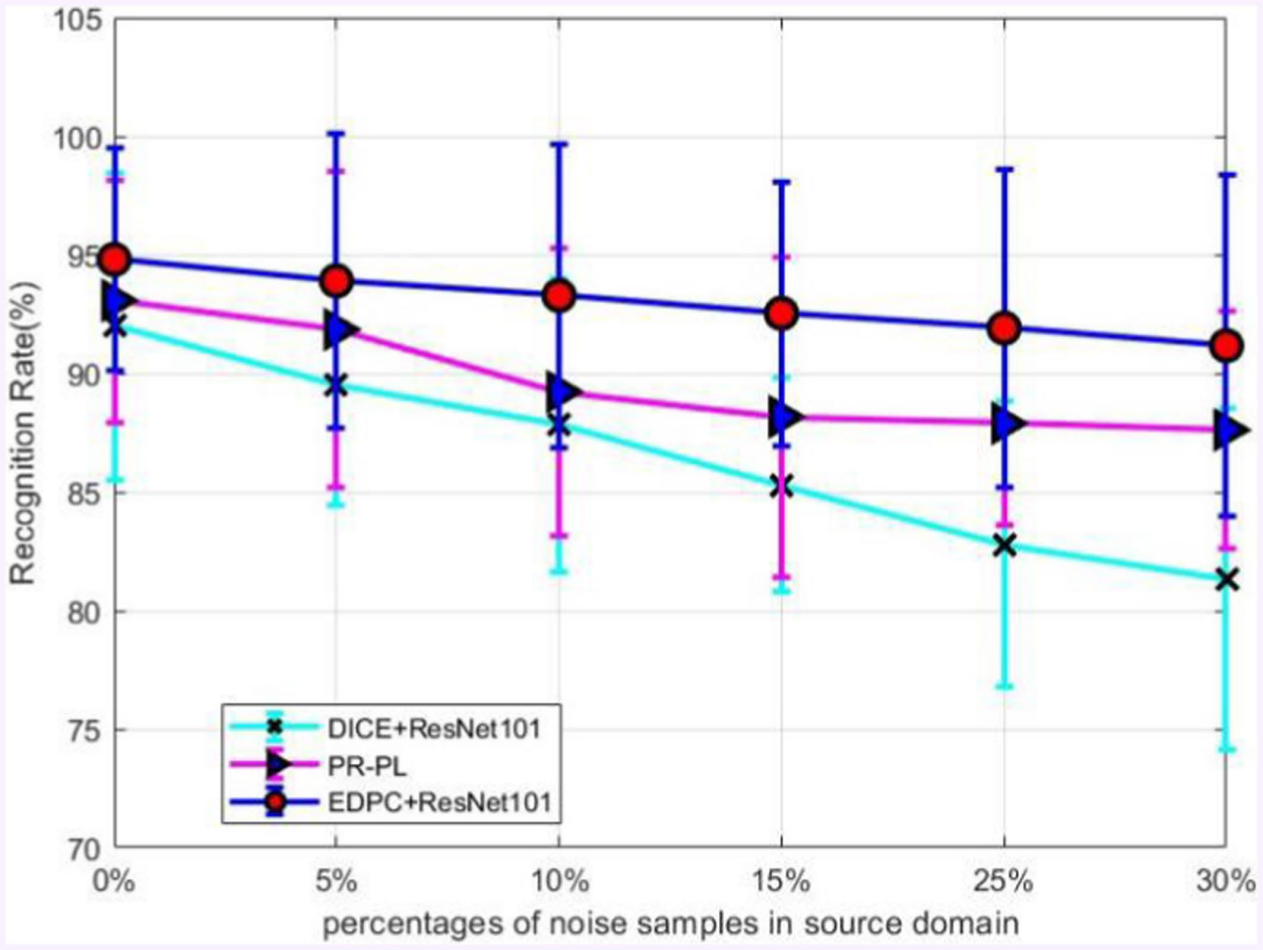
Robustness on source domain with different noise levels.

The experimental results shown in Figure 2 indicate that as the amount of noise increases, the accuracy of the proposed EDPC decreases at the slowest rate, demonstrating that EDPC is a reliable model with a high tolerance for noisy data. In future work, recent methods such as [52, 53] could be integrated to further reduce the more prevalent noise in EEG signals and enhance the model’s stability in cross-corpus applications.

### 6.3 Confusion matrices

**Figure 3:**
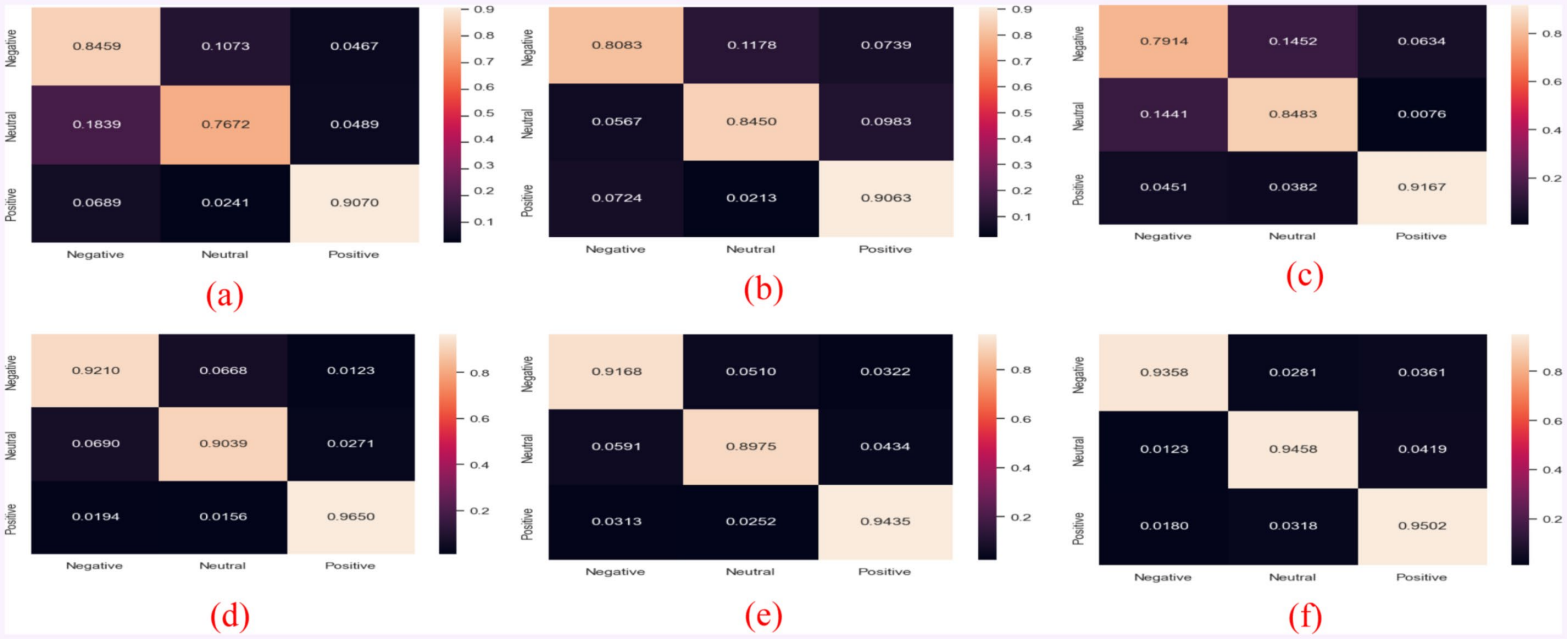
Confusion matrices of different models: (a) BiDANN; (b) BiHDM; (c) RGNN; (d) PR-PL; (e) DICE+ResNet101; and (f) EDPC+ResNet101.

To qualitatively study the performance of the model in each emotion category, this section visualizes the confusion matrix and compares the results with state-of-the-art models (i.e., BiDANN, BiHDM, RGNN, PR-PL, DICE ResNet101). As shown in the Figure 3, all models excel at distinguishing positive emotions from other emotions (with recognition rates above 90%), but they perform relatively poorly in distinguishing negative emotions from neutral emotions. For example, the emotion recognition rate in RGNN [25] is even below 80% (specifically, 79.14%).

Additionally, the PR-PL method slightly outperforms the EDPC method in recognizing positive emotions, likely due to the use of adversarial networks in PR-PL, which also increases its computational cost. Compared to existing methods (Figures 3a–c,e), the EDPC method is optimal, particularly in distinguishing neutral and negative emotions (even surpassing PR-PL). Furthermore, the overall performance of this method is superior to the DICE method (as shown in the comparison between Figures 3e,f).

### 6.4 Convergence

**Figure 4:**
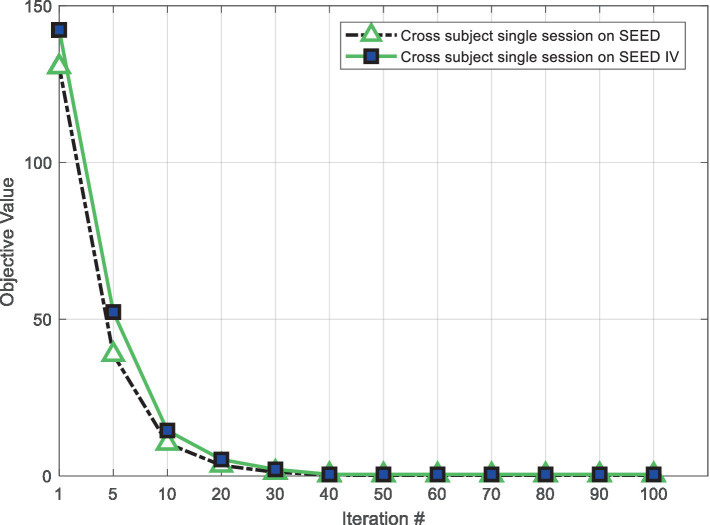
Convergence.

The proposed algorithm in this paper employs an iterative optimization strategy. To demonstrate the convergence of the algorithm, experiments were conducted on the MATLAB platform. The hardware configuration used for implementation includes 64GB of memory, a 2.5GHz CPU, and an 8-core Intel i7-11850H processor. The Figure 4 shows the convergence process of EDPC at different iteration counts. From the displayed results, it is evident that the proposed algorithm approaches convergence at around 30 iterations. In the algorithm, the objective function of each sub-problem optimization is a decreasing function, thereby proving that the EDPC method has good convergence properties.

## 7 Conclusion

The article analyzes the issue of mean shift that may arise during distribution distance measure in domain adaptation due to potential noise in the field. It proposes a novel distribution distance measure method with DPC criterion. This method determines the membership degree of each instance (i.e., the closer the distance, the more likely it is non-noise data) by measuring the distance between each instance and the overall domain mean. It uses membership degree and fuzzy trimming to mitigate the impact of noise data on final performance. Additionally, an outlier repulsion term is added during the domain adaptation process to further improve classifier discrimination accuracy. Based on this, a Emotion recognition DA method based on DPC is proposed (namely EDPC). It minimizes domain distribution differences while introducing a graph Laplacian matrix to learn a target domain label matrix and minimizes domain discrimination differences to learn a classifier for both the source and target domains. Theoretically, it is proven that the proposed algorithm has consistent convergence and an effective generalization error bound. Finally, the proposed EDPC method is extensively compared with the latest shallow and deep DAL methods on real datasets, validating its robustness and classification accuracy. Existing research results indicate that multi-source domain adaptation can effectively avoid the “negative transfer” situation caused by a single source domain. However, multi-source domain adaptation increases computational complexity. Therefore, constructing the EDPC model based on multi-source domain adaptation is a direction worthy of further research in this article.

## Data Availability

The raw data supporting the conclusions of this article will be made available by the authors, without undue reservation.

## Footnotes

^1^When n≠m , we can draw an equal number of samples from the source domain and the target domain, respectively.
